# Development of an Equivalent Circuit to Analyze the Receiving Characteristics of a Class IV Flextensional Transducer

**DOI:** 10.3390/s25185661

**Published:** 2025-09-11

**Authors:** Eunseo Kang, Yongrae Roh

**Affiliations:** School of Mechanical Engineering, Kyungpook National University, Daegu 41566, Republic of Korea; kang4ig@knu.ac.kr

**Keywords:** class IV flextensional transducer, hydrophone, equivalent circuit, end-plate, finite element method, receiving voltage sensitivity

## Abstract

Flextensional transducers are widely utilized as underwater acoustic transducers with broadband and high-sensitivity characteristics in low-frequency ranges. In this study, we have developed an equivalent circuit to facilitate the design of a class IV flextensional hydrophone. The end-plate of the class IV transducer is essential for sustaining the structure while keeping the hydrophone waterproof in underwater environments, and the presence or absence of the end-plate changes the hydrophone’s receiving performance. Previous studies have not included the end-plate in their equivalent circuit configurations, but this study proposes a new equivalent circuit model that incorporates an elastic boundary condition representing the constraint imposed on the shell by the compressive force of the end-plate. The receiving voltage sensitivity, calculated using the proposed equivalent circuit model, shows a high degree of agreement with the finite element analysis results, confirming that the mechanical influence of the end-plate significantly affects the hydrophone’s acoustic receiver characteristics. The proposed equivalent circuit approach offers faster computation while maintaining sufficient accuracy compared to the finite element analysis, making it a useful tool for future hydrophone design.

## 1. Introduction

A flextensional transducer (FT) operates through the coupling of longitudinal vibration in a driving element and flexural vibration of a shell. Depending on the shape and operating principle of the shell, FTs are divided into several classes and are widely used as underwater projectors or hydrophones [1]. When used as a transmitter, an FT efficiently converts longitudinal vibrations of piezoelectric ceramics into flexural deformation of the shell, resulting in high energy conversion efficiency in low-frequency ranges [2]. In particular, the class IV FT, which employs an elliptical shell, exhibits excellent acoustic performance and is therefore one of the most actively studied types of FTs [3].

To date, most studies have focused on class IV FTs for transmission applications, aiming to identify the variables affecting transducer performance and to design the class IV structures that meet desired specifications. Guo et al. proposed a new shell design to enhance the performance of class IV FTs [4]. Fang et al. adjusted the prestress applied to the shell to control the acoustic characteristics of the transducer [5]. Meanwhile, Zhou et al. modified a driving element so that the displacement could match that of the shell, thereby achieving broadband performance [6]. Li et al. investigated the methods to broaden transmission bandwidth by adjusting the curvature of the shell [7]. Feeney et al. miniaturized the class IV FT, achieving superior performance relative to the volume of piezoceramic and expanding its potential applications across various fields [8]. The findings from these studies on transmitting class IV FTs are also highly relevant for the development of class IV flextensional hydrophones (FHs) due to the reversibility of the piezoelectric effect.

However, despite this potential, studies analyzing the performance of class IV FHs remain very limited. Hydrophones, which detect underwater acoustic signals and convert them into electrical signals, play a crucial role in various applications such as underwater acoustic surveillance systems and marine exploration. Although research on class IV FHs reported to date is scarce, several notable studies have been conducted. For example, Varadan et al. analyzed the receiving sensitivity of FHs, but not specifically class IV, and investigated how design variables affect their receiving characteristics [9]. Zhang et al. proposed an FH design for high sensitivity in low-frequency ranges, but the shell structure was not clearly specified, nor did it conform to the class classification [10]. Garrett et al. designed a hydrophone using an elliptical shell structure similar to class IV, but employed a fiber-optic sensing method instead of electrical output, and the structure did not fully correspond to class IV [11]. Recent studies have reported that class IV FH exhibits the best acoustic receiving performance among various classes of FHs for receiving applications [12]. This suggests that the class IV structure can be effectively utilized as a hydrophone. Based on this, the present study aims to analyze the acoustic receiver characteristics of class IV FHs.

To precisely analyze the acoustic characteristics of transducers, previous studies have widely employed the finite element method (FEM). For example, Wang et al. analyzed the transmitting characteristics of class IV FTs using the commercial FEM software COMSOL^®^ [13]. Zheng et al. employed an optimal design process using the commercial FEM software ANSYS^®^ to design class IV FTs that meet target performance requirements, while Kim et al. utilized PzFlex^®^ (version 2018) for their analysis [12,14]. As such, designing transducers to achieve desired performance typically requires iterative analyses with various design parameters [15]. While FEM offers high accuracy in analysis results, it is computationally demanding. In that sense, the equivalent circuit method (ECM) is an alternative analysis approach to address these limitations. ECM analyzes a mechanical system by converting it into an equivalent electrical circuit with analogous characteristics, rather than directly analyzing the mechanical system. By simplifying mechanical systems into circuits, ECM offers a relatively straightforward calculation process and significantly reduces computation time compared to the FEM. Consequently, ECM enables rapid calculation of the transducer’s frequency response for various parameter changes and enhances understanding of the analysis results through physical intuition. Equivalent circuit models for class IV FTs have been studied as follows.

First, Debus et al. calculated the resonant frequencies of each shell mode by employing an equivalent circuit in which the longitudinal mode of the ceramic stack and the first and second flexural modes of the shell were each represented as motional branches [16]. Oswin also constructed a lumped equivalent circuit for class IV FTs to calculate the resonant frequencies of individual modes [17]. While these studies allow for the derivation of the resonant frequencies of class IV FTs through simple calculations, the equivalent circuits are only valid near resonance, excessively simplify the complex displacements of the shell, and include only the shell and ceramic components, limiting their practicality and realism for actual transducer design. In addition, Brigham theoretically formulated the displacement of a class IV shell by modeling it as a thin elliptical ring [18]. While this study is significant in that it provides an analytic form of the acoustic properties of class IV shells, its limitation is that the model is restricted to the shell part and does not describe the entire transducer. In a subsequent study, Brigham analyzed differential equations for the displacement of the elliptical shell and constructed an equivalent circuit for the class IV FT, including a ceramic rod and elliptic radiation function [19]. However, the shell model did not incorporate boundary conditions reflecting the installation state, resulting in calculation conditions that differ from the actual operating environment of the transducer. In reality, both ends of the shell are fixed and subjected to prestress, so the shell ends do not satisfy free boundary conditions. Building on Brigham’s work, Lam further simplified the calculations by assuming a constant thickness for the elliptical ring representing the shell [20]. Unlike Brigham, who included inserts within the shell, Lam separately considered the shell of constant thickness and inserts supporting the ceramic stack as distinct components. However, like Brigham, Lam also did not incorporate boundary conditions reflecting the installation state of the shell and limited the analysis to impedance calculations, lacking a comprehensive acoustic performance analysis for FTs specialized for either transmission or reception.

The end-plate supporting the structure of a class IV FT is a structural component essential for waterproofing during underwater operation, and it must be employed to provide protection against external water pressure as well as to maintain the transducer’s structural stability. From a mechanical perspective, the end-plate is attached to both ends of the shell, establishing compression-based boundary conditions that directly influence the vibration characteristics of the transducer. In practical underwater environments, the presence of the end-plate is essential. Thus, analytical models that omit the end-plate inevitably diverge from real-world conditions and may yield inaccurate predictions.

In conclusion, previous class IV FT equivalent circuit models considered only the shell and the PZT stack, neglecting essential structural components such as the end-plate and protective coating. This omission led to limited accuracy and applicability in practical underwater conditions, as these components significantly affected the mechanical and waterproofing behavior of the transducer. In contrast, our model explicitly includes these elements, thereby providing a more comprehensive and realistic representation of class IV FTs.

In this work, an elastic boundary condition reflecting the compressive force exerted by the end-plate is introduced into the equation of motion for a class IV FT shell. Based on this, a new equivalent circuit model is established for calculating the receiving voltage sensitivity (RVS) of a class IV FH. The equivalent circuit consists of the main components of a class IV FT, which are the mechanical and electrical impedances of the shell, insert, and piezoceramic. The influence of the end-plate was incorporated into the shell impedance. The RVS of a hydrophone is a key acoustic parameter representing the hydrophone’s sensitivity to external acoustic pressure. It serves as the foundation for calculating various acoustic characteristics required in hydrophone design, such as receiving bandwidth, peak RVS frequency, and fractional bandwidth. Using the derived equivalent circuit, the RVS of the class IV FH is calculated and validated through comparison with those obtained using a commercial finite element analysis (FEA) software PZFlex^®^ (version 2018).

## 2. Equivalent Circuit of the Class IV FH

**Figure 1:**
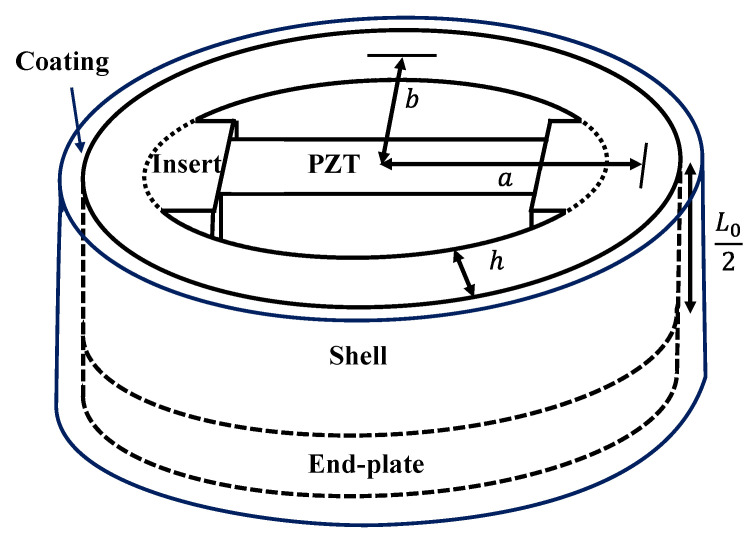
Schematic structure of the class IV FH.

**Figure 2:**
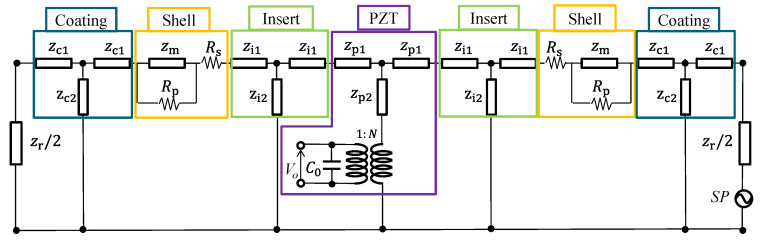
Equivalent circuit of a class IV FH for receiving operation.

A class IV FH features a structure in which both ends of a prismatic PZT ceramic stack are supported by inserts and closed by an elliptical shell. The ends of the shell are sealed with end-plates to prevent water ingress. The elliptical shell deforms when external acoustic pressure is applied, which leads to mechanical strain in the PZT ceramic. By measuring the voltage induced in the PZT ceramic by this deformation, the class IV FH functions as a receiver. The structure and components of the class IV FH are illustrated in Figure 1, where the semi-major axis *a*, semi-minor axis *b*, shell thickness *h*, and shell height *L*_0_ are denoted. The figure presents a cross-sectional view cut along the symmetry plane to show the internal configuration of the class IV FH. In this study, a class IV FH with the described structure is modeled as an equivalent circuit using the sample dimensions specified in Table 1. The proposed equivalent circuit, depicted in Figure 2, consists of four main elements: PZT ceramic stack, insert, shell including the end-plate, and radiation impedance. Impedance of the insert is represented as *z*_i1_ and *z*_i2_, while that of the ceramic stack is denoted as *z*_p1_ and *z*_p2_. The impedance of the coating is indicated as *z*_c1_ and *z*_c2_, and the capacitance and turns ratio of the piezoceramic stack are denoted as *C*_0_ and *N*, respectively. The radiation impedance is expressed as *z*_r_, and the mechanical impedance of the shell as *z*_m_. To account for damping effects, a series resistor *R*_s_ and a parallel resistor *R*_p_ are added to the shell impedance. External acoustic pressure *P* is defined as the force *F* applied per hydrophone’s surface area *S*, and the voltage induced in the piezoceramic stack is denoted as *V*_0_. Section 2.1, Section 2.2 and Section 2.3 detail the derivation of impedances for each component of the equivalent circuit, followed by a step-by-step procedure for calculating the RVS of the class IV FH using the proposed equivalent circuit.

### 2.1. Shell and End-Plate

The shell of the class IV FH is ring-shaped with an elliptical cross-section and is subjected to compressive force at both ends by the end-plates. This mechanical constraint can be reflected in the shell’s equation of motion by applying boundary conditions. In this section, the process of deriving the mechanical impedance of the shell, incorporating the effect of the end-plate as an elastic boundary condition, is described.

**Figure 3:**
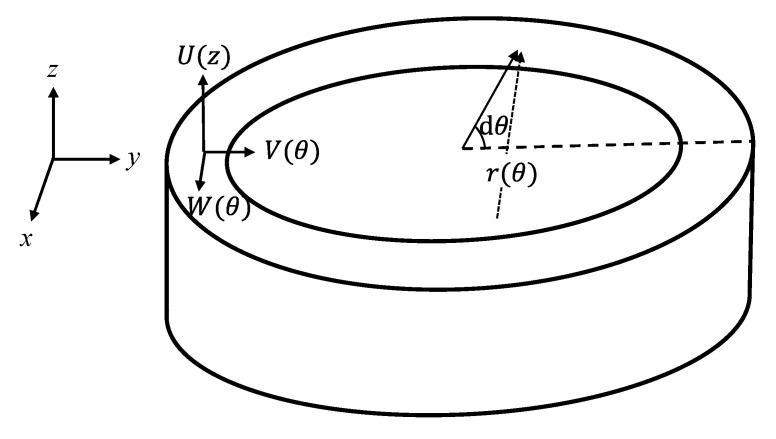
Coordinate of the class IV FH shell model.

The equation of motion for the elliptical shell of the class IV FT is given by Equation (1) [16]. This represents the balance of forces acting on the shell in both the radial and tangential directions. Here, the density and thickness of the shell are denoted by *ρ*_s_ and *h*_s_, respectively. As shown in Figure 3, the product of the shell’s radius of curvature *r* and the angle *dθ* from the ellipse center corresponds to the differential arc length *ds* of the ellipse. *T*_s_ represents the tensile stress acting in the z-axis direction, and *N*_s_ denotes the shear stress acting perpendicular to the XY plane. *P*^N^ and *P*^T^ represent the external pressures normal and tangential to the shell surface, respectively.

(1) $$\frac{T_{s}}{r}+\frac{\partial N_{s}}{\partial s}=\rho_{s}h_{s}\frac{\partial^{2}V}{\partial t^{2}}+P^{N}\;\frac{\partial T_{s}}{\partial s}-\frac{N_{s}}{r}=\rho_{s}h_{s}\frac{\partial^{2}W}{\partial t^{2}}+P^{T}\;\mathrm{where}\;\mathrm{ds}=r\;d\theta$$

The radial displacement *V*, tangential displacement *W*, and axial displacement *U* of the shell, which satisfy the equation of motion, are expressed in the modal superposition forms shown in Equation (2) [17]. Since *V* and *W* always have a phase difference of 90°, they are represented by cosine and sine functions, respectively, to reflect the relationship between the two variables. These displacements are expressed as Fourier series to capture the periodic displacement characteristics of the shell, where *V*_2j_ and *W*_2j_ in Equation (2) denote the amplitudes of the j-th mode displacement. In contrast, since *U* does not vary around the shell’s circumference, it is modeled as a linear function proportional to the axial coordinate z of the shell.

(2) $$V=\sum_{j=1}^{m}V_{2j}\cos2j\theta \;W=\sum_{j=1}^{m}W_{2j}\sin2j\theta \;U=U_{0}z$$

Assuming that *V* and *W* include a time-harmonic term $e^{i\omega t}$, the equation of motion in Equation (1) can be expressed as Equation (3), where *ω* is the angular frequency. Substituting the assumed solution form from Equation (2) into *T*_s_ and *N*_s_ leads to Equation (4). Ultimately, the equation of motion can be represented in terms of the displacements *V* and *W,* as shown in Equations (5) and (6). To calculate the coefficients *Q*^N^_2l,2j_, *K*^N’^_2l,2j_, *C*^N^_2l_, *M*^N^_2l,2j_, *Q*^T^_2l,2j_, *K*^T’^_2l,2j_, and *M*^T’^_2l,2j_, each expression in Equation (3) is multiplied by cos 2lθ and sin 2lθ, respectively, utilizing orthogonality. These coefficients are non-zero only when *l* = *j*, where *l* is a term index. Additionally, *E*_s_ and *σ*_s_ represent Young’s modulus and Poisson’s ratio of the shell material, respectively.

(3) $$\frac{T_{s}}{r}+\frac{\partial N_{s}}{\partial s}=-\omega^{2}\rho_{s}h_{s}V+P^{N}\;\frac{\partial T_{s}}{\partial s}-\frac{N_{s}}{r}=-\omega^{2}\rho_{s}h_{s}W+P^{T}$$

(4) $$T_{s}=\frac{E_{s}h_{s}}{1-\sigma_{s}^{2}}(\frac{\partial W}{\partial s}-\frac{V}{r}+\sigma_{s}U_{0})-\frac{D}{2r\left(1-\sigma_{s}\right)}(2+\sigma_{s})\frac{\partial }{\partial s}(\frac{\partial V}{\partial s}+\frac{W}{r})\;N_{s}=-\frac{E_{s}h_{s}^{3}}{12(1-\sigma_{s}^{2})}\frac{\partial^{2}}{\partial s^{2}}(\frac{\partial V}{\partial s}+\frac{W}{r})$$

(5) $$\sum_{j=1}^{m}(2{jW}_{2j}-V_{2j})Q_{2l,2j}^{N}-\sum_{j=1}^{m}(W_{2j}-2{jV}_{2j})K_{2l,2j}^{N\prime }+C_{2l}^{N}=-\omega^{2}\sum_{j=1}^{m}M_{2l,2j}^{N}V_{2j}+P_{2l}^{N\prime }\;\mathrm{where}Q_{2l,2j}^{N}=\int_{0}^{2\pi }\frac{E_{s}h_{s}}{1-\sigma_{s}^{2}}\frac{\cos\;2j\theta \;\cos\;2l\theta }{r}d\theta \;K_{2l,2j}^{N\prime }=-D\int_{0}^{2\pi }\frac{2j\;\cos\;2j\theta -\alpha \;\sin\;2j\theta }{r^{2}}(\frac{\partial^{2}\;\cos\;2l\theta }{\partial s^{2}}+\frac{2+\sigma_{s}}{2r(1-\sigma_{s})}\cos\;2l\theta )d\theta \;D=\frac{E_{s}h_{s}^{3}}{12(1-\sigma_{s}^{2})},\;\alpha =\frac{1}{r}\frac{\mathrm{dr}}{d\theta }\;C_{2l}^{N}=\int_{0}^{2\pi }\frac{E_{s}h_{s}}{1-\sigma_{s}^{2}}\frac{\sigma_{s}U_{0}}{r}\cos\;2l\theta \;d\theta \;M_{2l,2j}^{N}=\int_{0}^{2\pi }\rho_{s}h_{s}\cos\;2j\theta \;\cos\;2l\theta \;d\theta$$

(6) $$\sum_{j=1}^{m}(V_{2j}-2{jW}_{2j})Q_{2l,2j}^{T}+\sum_{j=1}^{m}(W_{2j}-2{jV}_{2j})K_{2l,2j}^{T\prime }=-\omega^{2}\sum_{j=1}^{m}M_{2l,2j}^{T\prime }W_{2j}+P_{2l}^{T}\;\mathrm{where}Q_{2l,2j}^{T}=\int_{0}^{2\pi }\frac{E_{s}h_{s}}{1-\sigma_{s}^{2}}\frac{2j\;\sin\;2j\theta +\alpha \;\cos\;2j\theta }{r^{2}}\sin\;2l\theta \;d\theta \;K_{2l,2j}^{T\prime }=-\frac{D(2+\sigma_{s})}{2(1-\sigma_{s})}\int_{0}^{2\pi }\frac{2j\;\cos\;2j\theta -\alpha \;\sin\;2j\theta }{r^{3}}(\frac{\alpha \;\sin2j\theta }{r}+\frac{4-\sigma_{s}}{2+\sigma_{s}}\frac{\partial \sin2l\theta \;}{\partial s})d\theta \;M_{2l,2j}^{T\prime }=\int_{0}^{2\pi }\rho_{s}h_{s}\;\sin\;2j\theta \;\sin\;2l\theta \;d\theta$$

When a class IV FH operates, deformation in the shell’s thickness direction is negligible, or, if present, is minimal compared to the overall shell deformation. By neglecting the deformation in the thickness direction, the relationship *V*_2j_ = 2*jW*_2j_ holds, allowing the entire equation to be simplified as a function of *V* alone. Accordingly, the coefficients are developed in the form shown in Equation (7). For efficient computation, each coefficient term is organized in matrix form as shown in Equation (8). If the number of Fourier terms included in the calculation is denoted as *m*, then ***Q*^N^**, ***Q*^T^**, ***K*^N^**, ***K*^T^**, ***M*^N^**, and ***M*^T^** are all constructed as *m* × *m* matrices, following the same approach as ***Q*^N^**, while ***V***, ***P*^N^**, and ***P*^T^** are formed as *m* × 1 vectors, consistent with ***V***. Since the calculated ***V*** matrix contains the undetermined coefficient *U*_0_, it is necessary to apply boundary conditions to determine *U*_0_.

(7) $$P_{2l}^{N}=P_{2l}^{N\prime }-C_{2l}^{N}\;K_{2l,2j}^{N}=\frac{4j^{2}-1}{2j}K_{2l,2j}^{N\prime }\;K_{2l,2j}^{T}=\frac{1-4j^{2}}{2j}K_{2l,2j}^{T\prime }\;M_{2l,2j}^{T}=\frac{1}{2j}M_{2l,2j}^{T\prime }$$

(8) $$[(Q^{N}{)}^{-1}K^{N}+(Q^{T}{)}^{-1}K^{T}-\omega^{2}\{(Q^{N}{)}^{-1}M^{N}+(Q^{T}{)}^{-1}M^{T}\}]V=-(Q^{N}{)}^{-1}P^{N}+(Q^{T}{)}^{-1}P^{T}\;\mathrm{where}\;Q^{N}\;=\;\left[\begin{array}{ccc} Q_{2,2}^{N} & \ldots & Q_{2,2m}^{N}\\ \vdots & \ddots & \vdots \\ Q_{2m,2}^{N} & \ldots & Q_{2m,2m}^{N} \end{array}\right],\;V\;=\;\left[\begin{array}{c} \begin{array}{c} V_{2}\\ \vdots \end{array}\\ V_{2m} \end{array}\right]$$

The effect of the end-plate is represented by an elastic boundary condition. It is assumed that an elastic force *F*_b_ acts on the boundary surface of the shell due to a spring constant *k*_b_, and the elastic stress *T*_b_ is defined based on the area *A* on which the force acts. The related relationship is presented in Equation (9). Here, the spring constant *k*_b_ is not a physical property of an actual structure but is introduced as a virtual parameter in the equivalent circuit.

(9) $$F_{b}=-k_{b}U_{0}z\;T_{b}=-\frac{F_{b}}{A}\;A=\pi (a+\frac{h}{2})(b+\frac{h}{2})-\pi (a-\frac{h}{2})(b-\frac{h}{2})$$

The exact value of the parameter *k*_b_ is calibrated through comparison of the RVS spectrum calculated by the equivalent circuit with that obtained by FEM in a later section. The elastic stress *T*_b_ generated by the force of the end-plate acts on the shell’s boundary surfaces at $z=\pm \frac{L_{0}}{2}$, and thus the boundary condition is given in Equation (10). *U*_0_ is obtained by applying this boundary condition and substituting into the solution ***V*** from Equation (8), yielding the radial displacement ***V***_F_ of the shell. The tangential displacement ***W***_F_ is then determined by ***V***_F_, and the total displacement ***V***_C_, which includes both the radial and tangential components, is expressed as shown in Equation (11). Here, ***V***_F,2l_ denotes the 2*l*-th term of ***V***_F_.

(10) $$T_{s}{|}_{z=\pm \frac{L_{0}}{2}}=T_{b}$$

(11) $$V_{c}=\sum_{i}\int_{-\theta_{c}}^{\theta_{c}}V_{F,2l}\frac{\left(\cos\;\theta \;\cos2l\theta +\frac{\sin\theta \;\sin2l\theta }{2l}\right)}{L_{0}}r\;d\theta$$

The mechanical impedance of the shell, *z*_m_, is defined as the ratio of the external force *F* applied to the shell to the vibration velocity *u* of the shell, as shown in Equation (12) [17]. For this calculation, a unit force *F* = 1 is assumed, and the velocity *u* is obtained by differentiating the displacement ***V***_C_ with respect to time, yielding *iω**V***_C_. Thus, the mechanical impedance is calculated as described in Equation (12).

(12) $$z_{m}=\frac{F}{u}=\frac{1}{i\omega V_{C}}$$

**Figure 4:**
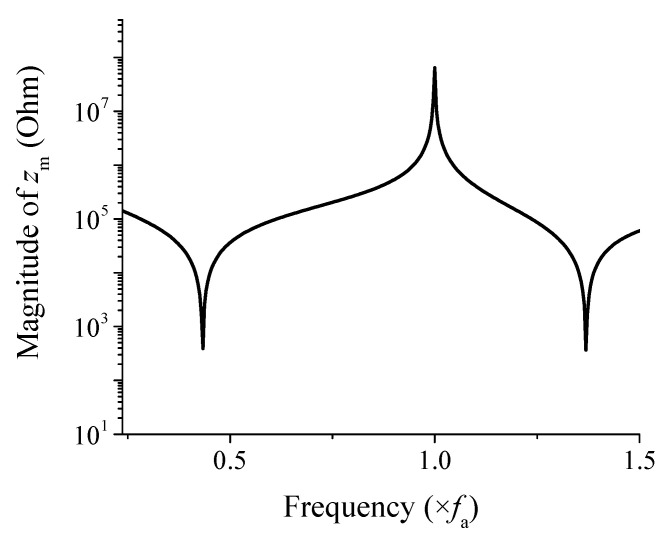
Spectrum of the shell impedance magnitude, |*z*_m_|.

Figure 4 shows the variation of the magnitude of *z*_m_ as a function of frequency when the shell material is aluminum [21]. The horizontal axis was normalized to the first peak frequency *f*_a_ in the spectrum. To normalize the horizontal axis, all frequency values in the spectrum were divided by the first peak frequency *f*_a_. This means that the first peak frequency corresponds to 1 on the horizontal axis after normalization. Since the structure of the FH is symmetric, the calculated shell impedance is equally divided into the left and right sides in the equivalent circuit. However, the calculated *z*_m_ does not include the components to represent the damping effect of the shell. To reflect actual operating conditions more accurately, *R*_p_ and *R*_s_ are added to *z*_m_/2 in the equivalent circuit. By incorporating these resistive elements, the corrected shell impedance is defined as *z*_md_.

### 2.2. Piezoceramic Stack, Insert, and Coating

When a class IV FH operates, external acoustic pressure causes its shell to deform, which in turn induces longitudinal vibration of the piezoceramic stack. The equivalent circuit for the piezoceramic stack, as illustrated in Figure 2, consists of an electrical capacitance and a T-network representing its mechanical characteristics. The turns ratio *N*, mechanical impedances *z*_p1_, *z*_p2_, and capacitance *C*_0_ of the longitudinally vibrating PZT ceramic stack are calculated as shown in Equation (13) [21]. Here, *ρ*_p_, *c*_p_, *A*_p_, *L*_p_, and *k*_p_ denote the density, speed of sound, cross-sectional area, length, and wavenumber of the ceramic stack, respectively. *d*_33_, *s*^E^_33_, and *ε*^S^_33_ represent the piezoelectric strain constant, elastic compliance at constant electric field, and permittivity at constant strain, all along the poled direction of the piezoceramic, respectively. PZT-4 was used for the piezoceramic [21].

(13) $$N=\frac{A_{p}d_{33}}{L_{p}\;s_{33}^{E}}\;C_{0}=\frac{\varepsilon_{33}^{S}A_{p}}{L_{p}}\;z_{p1}={iz}_{p}\tan\;\frac{k_{p}L_{p}}{2}\;z_{p2}=\frac{-{iz}_{p}}{\sin\left(k_{p}L_{p}\right)}\;\mathrm{where}\;z_{p}=\rho_{p}\;c_{p}\;A_{p}$$

Inserts serve as a structural component that supports the PZT ceramic and connects it to the shell in a class IV FH. They are made of aluminum, the same material as that of the shell. Since aluminum is not a piezoelectric material, inserts can be represented as a simple T-network in the equivalent circuit, as shown in Figure 2. The impedance values for the elements of the insert’s T-network are given in Equation (14). Here, *ρ_i_*, *c*_i_, *A*_i_, *L*_i_, and *k*_i_ denote the density, speed of sound, cross-sectional area, length, and wavenumber of the insert, respectively. Since inserts are attached to both ends of the PZT ceramic, the equivalent circuit of the piezoceramic is placed at the center, with T-networks of the inserts connected on both sides.

(14) $$z_{i}=\rho_{i}c_{i}A_{i}\;z_{i1}={iz}_{i}\tan(\frac{k_{i}L_{i}}{2})\;z_{i2}=\frac{-{iz}_{i}}{\sin\left(k_{i}L_{i}\right)}$$

The coating is the final component added to the exterior of a class IV FH to protect the hydrophone from water. This coating is made of rubber, which is a non-piezoelectric material. Therefore, it is modeled as a T-network in the equivalent circuit, and the impedance values for its elements are summarized in Equation (15). Here, *ρ_c_*, *c*_c_, *A*_c_, *L*_c_, and *k*_c_ represent the density, speed of sound, cross-sectional area, length, and wavenumber of the coating, respectively. The T-network representing the coating is positioned between the shell impedance and the radiation impedance in the equivalent circuit.

(15) $$z_{c}=\rho_{c}c_{c}A_{c}\;z_{c1}={iz}_{c}\tan(\frac{k_{c}L_{c}}{2})\;z_{c2}=\frac{-{iz}_{c}}{\sin\left(k_{c}L_{c}\right)}$$

### 2.3. Radiation Impedance

When a hydrophone receives underwater sound waves, a radiation impedance, which corresponds to the acoustic load from a propagation medium, must be included in a receiving equivalent circuit. The radiation impedance varies depending on the shape of a hydrophone’s surface, but if the size of the hydrophone is sufficiently small compared with the wavelength, the effect of the shape can be considered negligible. For the sake of computational simplicity, the elliptical shell is approximated as an ideal sphere, and the corresponding radiation impedance is applied as in Equation (16) [22]. Here, *ρ*_w,_ *c*_w,_ and *k*_w_ denote the density, speed of sound, and wavenumber of water, respectively. The radius *r*_s_ of the spherical source is calculated as the radius of a sphere having the same surface area *S* as the hydrophone.

(16) $$z_{r}=\rho_{w}c_{w}S\;\;\cos\;\theta_{r}(\cos\;\theta_{r}+i\;\sin\;\theta_{r})\;\mathrm{where}\;\tan\;\theta_{r}=\frac{1}{k_{w}r_{s}}$$

**Figure 5:**
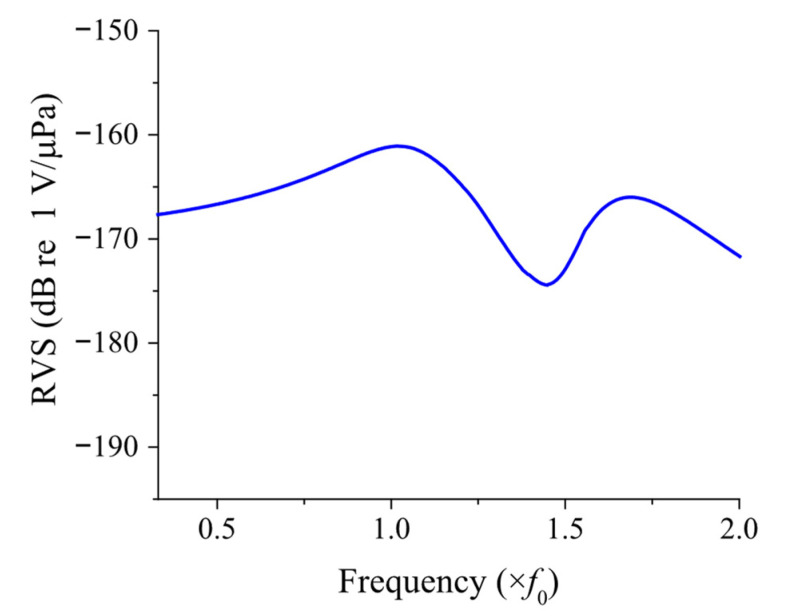
RVS spectrum of the class IV FH calculated by the equivalent circuit.

The RVS, representing the resultant performance of the hydrophone, can be calculated using the equivalent circuit shown in Figure 2. When an acoustic pressure *P* is applied over the surface area *S*, the voltage *V*_0_ induced across the electrodes of the piezoceramic stack is determined using the equivalent circuit. The RVS is then obtained by using Equation (17). The RVS spectrum of the sample FH with the dimensions given in Table 1 is presented in Figure 5, where the horizontal axis is normalized to the first peak RVS frequency, *f*_0_.

(17) $$\mathrm{RVS}=20\;\log(V_{0}/P)\;\;\;(\mathrm{dB}\;\mathrm{re}\;1\;V/\mu \mathrm{Pa})$$

## 3. Finite Element Analysis

**Figure 6:**
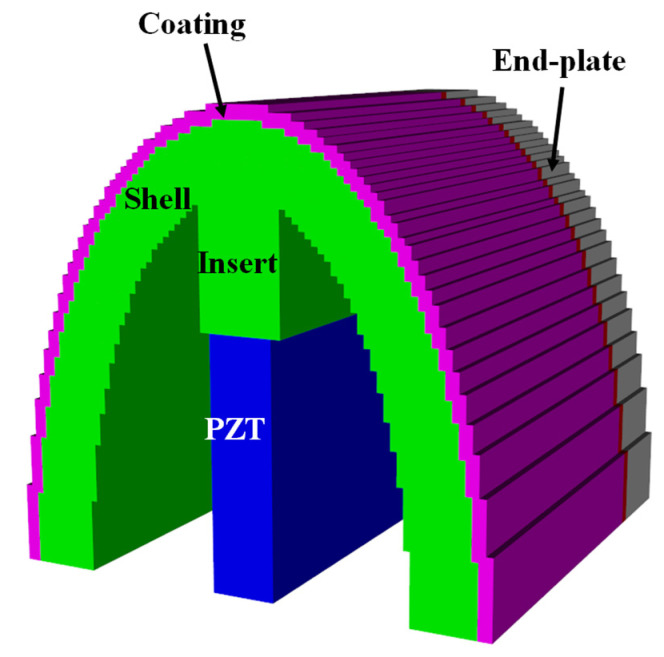
FEA Model of the class IV FH.

**Figure 7:**
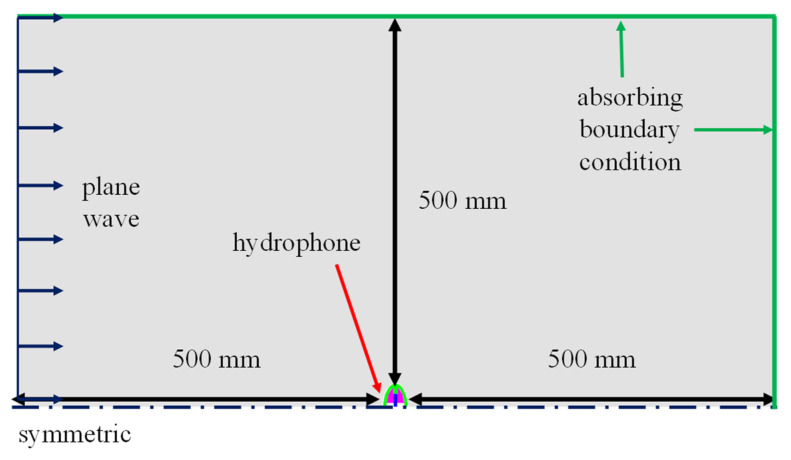
FEA Model to analyze underwater sound wave receiving performance of the class IV FH.

**Figure 8:**
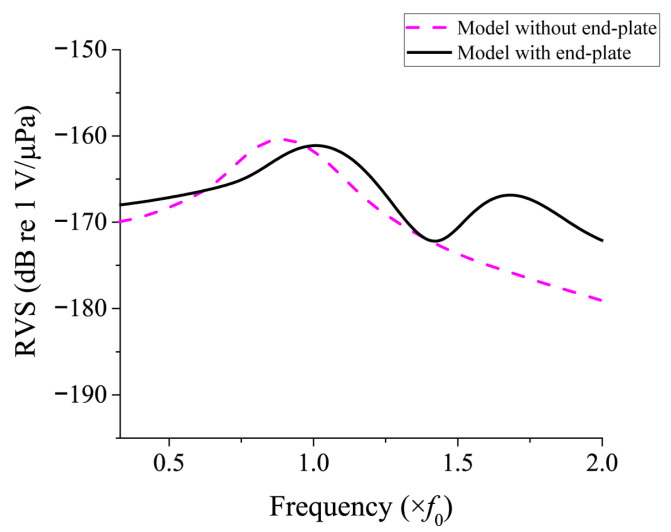
Comparison of the RVS spectra of the class IV FH with and without the end-plate.

Since the equivalent circuit proposed in this study is a new model, it is necessary to verify the validity of its analysis. For this purpose, the RVS of the class IV FH was calculated using the commercial FEA software PZFlex^®^, and the results were compared with those obtained from the proposed equivalent circuit analysis (ECA). Figure 6 shows the finite element model of the class IV FH, where only one-quarter of the entire structure was modeled considering symmetry. The dimensions of the model are identical to those set in the equivalent circuit. Figure 7 shows the model for analyzing the underwater acoustic receiving characteristics, in which a plane wave is incident along the minor axis direction of the hydrophone. Absorbing boundary conditions were applied to all surfaces of the water domain, except for the symmetry planes, to eliminate the influence of reflected waves. The entire model consisted of approximately 74 million elements and about 75 million nodes. The RVS spectrum obtained from the analysis is shown in Figure 8. Additionally, to confirm the effect of end-plates on the RVS of the hydrophone, the analysis results for a model without the end-plate under the same conditions are also presented in Figure 8. The model with the end-plate showed a higher first peak RVS frequency and introduced a new second peak that was not present in the model without the end-plate. These results confirm that the end-plate has a significant impact on the resonant characteristics of the FH and that unrealistic models that do not include the end-plate are likely to yield inaccurate results.

## 4. Model Validation and Parameter Sweep Analysis

### 4.1. Comparison of Results from ECA and FEA

To enhance consistency between the ECA and FEA, a calibration process was conducted on the variables of the equivalent circuit. Although the ECA offers higher computational efficiency than the FEA, the simplification in constructing the circuit makes it difficult to capture all the details of physical phenomena. Therefore, calibration with FEA results is necessary to ensure the reliability of the ECA.

The purpose of the calibration is to determine the spring constant *k*_b_ in Equation (9) and the resistors *R*_p_ and *R*_s_ in Figure 4, so that the primary characteristics of the RVS spectrum, the first and second peak frequencies, and the RVS levels at those frequencies, match the FEA results as closely as possible. To achieve this, the combination of these three parameters was optimized to minimize the objective function representing the difference between the ECA and FEA results. The objective function is presented in Equation (18), and no additional constraints were imposed. Here, *f*_c1_, *f*_c2_, *L*_c1_, and *L*_c2_ denote the first and second peak RVS frequencies and the RVS levels at those peak frequencies calculated by the ECM, while *f*_f1_, *f*_f2_, *L*_f1_, and *L*_f2_ represent those calculated by the FEM. The valley refers to the local minimum observed between the two peaks in the RVS spectrum. The RVS levels of the valley calculated by the ECM and FEM are denoted as *L*_c_ and *L*_f_, respectively. This objective function represents the normalized squared difference between the ECA and FEA results.

(18) $$\begin{array}{l} \mathrm{Objective}\mathrm{function}:\;\mathrm{Minimize}\\ \;\;\;\;\frac{{\left(f_{c1}-f_{f1}\right)}^{2}}{f_{c1}^{2}}+\frac{{\left(f_{c2}-f_{f2}\right)}^{2}}{f_{c2}}+\frac{{\left(L_{c1}-L_{f1}\right)}^{2}}{L_{c1}^{2}}+\frac{{\left(L_{c2}-L_{f2}\right)}^{2}}{L_{c2}^{2}}+\frac{{\left(L_{c}-L_{f}\right)}^{2}}{L_{c}^{2}} \end{array}$$

The parameters used to adjust the values of the objective function were limited to the spring constant *k*_b_ and resistors *R*_s_ and *R*_p_. All other structural parameters of the hydrophone, including its geometric and material properties, were set to be identical to those used in the FEA.

The sample analysis cases required in the optimization process were selected by the 3 k experimental design method [23]. The range of variation of the parameters is presented in Table 2. Analysis of the hydrophone performance showed that increasing both *k*_b_ and *R*_p_ led to an increase in the first and second peak RVS frequencies, while increasing *R*_s_ resulted in a decrease in both the peak frequencies. A regression analysis was performed on the simulation data. Based on these results, the optimal combination of *k*_b_, *R*_s_, and *R*_p_ that satisfied Equation (18) was determined using the OptQuest Nonlinear Programming (OQNLP) algorithm [24]. The optimal values for *k*_b_, *R*_s_, and *R*_p_ were found to be $1.29\times 10^{6}$ N/m, $1.08\times 10^{5}$ Ohm, $1.89\times 10^{5}$ Ohm, respectively.

**Figure 9:**
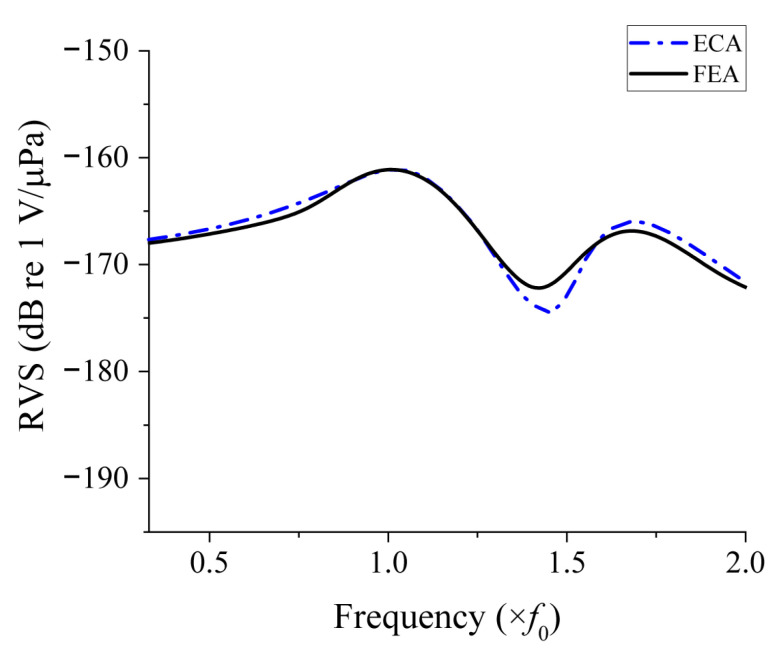
Comparison of RVS spectra of class IV FH by analysis methods.

The three parameters were incorporated into the equivalent circuit in Figure 2, and the RVS spectrum calculated with the equivalent circuit is compared with that from the FEA in Figure 9. Both spectra exhibited two distinct RVS peaks. The frequencies and levels of the two peaks are summarized in Table 3. The first peak frequencies obtained by the ECM and FEM differed by 1.0%, and the second peak frequencies by merely 0.8%, indicating good agreement. The first peak RVS level calculated by ECM and FEM differed by less than 0.1%, and the second peak RVS level differed by less than 0.5%, demonstrating very close correspondence. These results confirm that the proposed equivalent circuit provides accuracy comparable to that of the FEM for both the peak frequency and peak RVS level.

Although the accuracy of the ECA and FEA was comparable, as shown in Table 3, there was a significant difference in the time required to calculate the RVS spectrum. The ECA took approximately 23 s, whereas the FEA under the same conditions required about 8 h, 30 min, and 8 s. By using the equivalent circuit, the RVS spectrum calculation was approximately 1330 times faster than using the FEM. This substantial reduction in analysis time facilitates the evaluation of hydrophone characteristics under various parameter variations during the design and structural optimization process of the FT, greatly improving overall design efficiency.

### 4.2. Parameter Sweep Analysis via the Equivalent Circuit Model

Using the equivalent circuit model validated in Figure 9, another example of parameter sweep analysis was conducted to investigate how variations in model parameters affected the RVS of the class IV FH. Equivalent circuit models enable parameter sweeps more effectively and efficiently than full FEM simulations by significantly reducing computation cost and time, while still providing accurate trend predictions.

**Figure 10:**
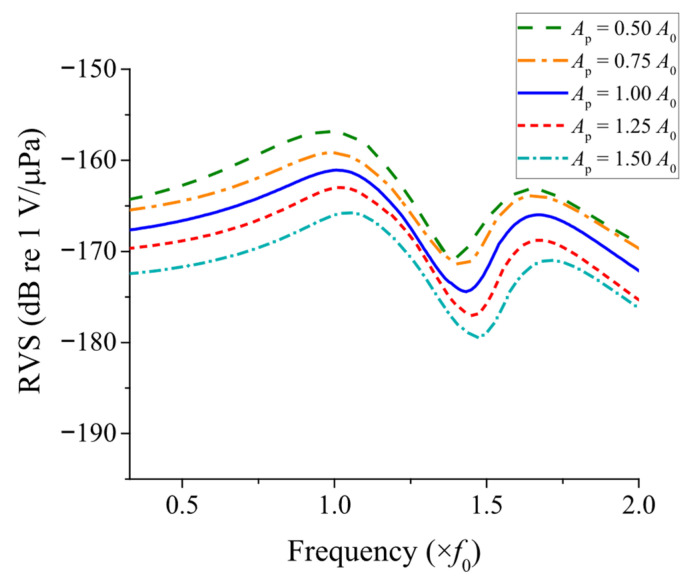
RVS spectra under different PZT area variations.

**Figure 11:**
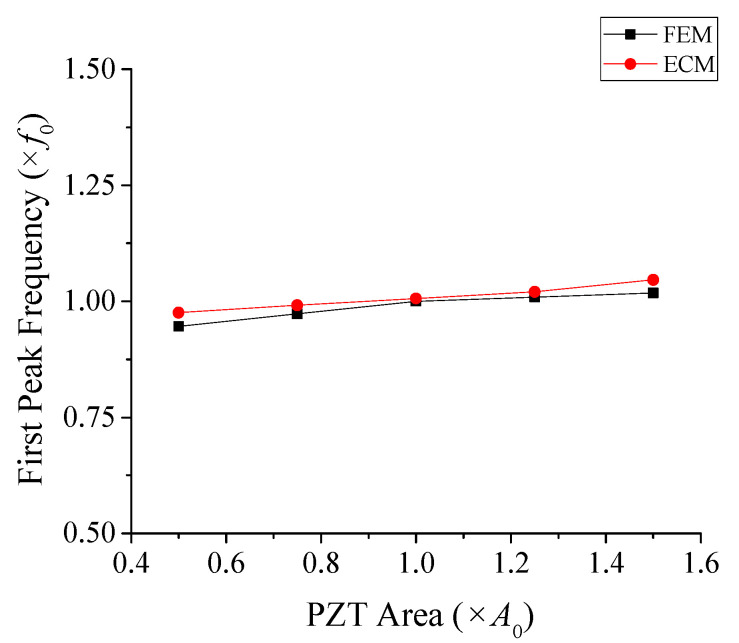
Effect of PZT area variation on the first peak frequency in the RVS spectrum.

In this analysis, the active area of the PZT stack was selected as the representative parameter due to its direct impact on the electromechanical coupling and overall sensitivity of the transducer. The cross-sectional area of the PZT in the model shown in Figure 9 was denoted as *A*_0_, and systematically varied by −50%, −25%, +25%, and +50% from *A*_0_, and the resulting RVS values were computed. The calculated RVS responses for these variations are shown in Figure 10. The variation of the first peak frequency with respect to the PZT area is plotted in Figure 11. The variation of the first peak frequency was also obtained by FEM and is compared in Figure 11. The results obtained by the ECM and FEM showed a good agreement with each other, with a difference of less than 3.1% within the PZT area variation range, which validated the ECA results. As the calibration in Section 4.1 was optimized for the reference condition when *A*_p_ = *A*_0_, the accuracy of the ECA naturally decreased as the model deviated further from the reference.

The level and frequency characteristics of the RVS response were influenced by the variation in the PZT area. First, the RVS level decreased with the enlargement of the PZT area. This relationship arises because the static capacitance *C*_0_ of the PZT stack, shown in Equation (19), is proportional to the PZT area *A*_p_ [25]. The voltage across the PZT stack, *V*_0_, is inversely related to its capacitance. Therefore, as the PZT area *A*_p_ increases, the capacitance increases proportionally, leading to a reduction in *V*_0_. Consequently, according to Equation (17), this results in a lower RVS level.

(19) $$C_{0}=\varepsilon_{33}^{S}\frac{A_{p}}{L_{p}}$$

Additionally, the anti-resonant frequencies of the peak RVS shifted as the PZT area varied. This shift is attributed to changes in the acoustic impedance of the PZT stack, which modifies the frequency response of the system.

This example demonstrates the efficacy of the developed equivalent circuit model as an efficient and insightful tool for trend and sensitivity analyses. It enables rapid exploration of design variations and facilitates the physical understanding of complex electromechanical interactions that would require significantly greater computational resources using FEM alone.

The nature of the ECM makes it feasible to conduct rigorous and accurate optimization. FEM-based optimization is limited by its high computational cost, i.e., required computation resources and time, often requiring only a small set of sampled simulations and relying on supplementary techniques such as regression analysis. These additional steps not only extend the total computational time but also introduce opportunities for error accumulation, potentially reducing the overall accuracy of the optimization. In contrast, the ECM enables rapid simulation of a large number of cases, facilitating direct and extensive optimization without the need for secondary methods like regression. This advantage further demonstrates the practical significance of the ECA approach for design exploration and optimization.

## 5. Conclusions

In this study, a new equivalent circuit was proposed to analyze the acoustic receiving characteristics of a class IV FH more accurately and efficiently. The end-plate of a class IV FH is essential for supporting the structure and waterproofing of the underwater transducer, and it also significantly affects the acoustic characteristics of the hydrophone. However, equivalent circuits reported to date for the class IV FH have not properly reflected the influence of the end-plate. In addition, previous studies have only analyzed up to impedance spectra and have not directly analyzed the acoustic receiving characteristics of the transducer as a hydrophone.

To derive more realistic and practical analytical results, we analyzed the vibration characteristics of the shell, including the influence of the end-plate, and incorporated them into the equivalent circuit. Furthermore, this study analyzed the RVS spectrum of the class IV FH, which represents the ultimate performance of the hydrophone with the proposed circuit. The validity of the developed equivalent circuit was demonstrated by comparing the RVS spectrum obtained from the circuit with that calculated by FEM, showing good agreement with each other. The proposed equivalent circuit maintains accuracy comparable to FEM while significantly reducing computation time to approximately 1/1330, thus enabling efficient analysis and design of a class IV FH. Further work will involve modifying and applying the current circuit to other classes of FTs to verify its scalability and generality. In addition, fabrication and experimental validation of a class IV flextensional hydrophone based on the results of this study are planned for future work.

## Figures and Tables

**Table 1:** Dimensions of the class IV FH model.

Structural Parameter	Symbol	Value [mm]
Semi-major axis length	*a*	29.4
Semi-minor axis length	*b*	14.5
Shell thickness	*h*	4.6
Shell height	*L*_0_	54.0

**Table 2:** Variation range of the calibration parameters in accordance with normalization factors.

	Normalization Factor	−1	0	1
Parameter				
*k*_b_ (N/m)		1.1 × 10^6^	1.2 × 10^6^	1.3 × 10^6^
*R*_s_ (Ohm)		5.0 × 10^4^	1.0 × 10^5^	1.5 × 10^5^
*R*_p_ (Ohm)		1.0 × 10^5^	2.0 × 10^5^	3.0 × 10^5^

**Table 3:** Comparison of the RVS spectrum analysis results by ECM and FEM.

Acoustic Property	ECA	FEA
1st peak frequency (×*f*_0_)	1.00	1.01
2nd peak frequency (×*f*_0_)	1.69	1.68
1st peak level (dB)	−161.3	−161.1
2nd peak level (dB)	−166.0	−166.8

## Data Availability

The original contributions presented in the study are included in the article; further inquiries can be directed to the corresponding author.
